# Supplementary material for: Prevalence of SARS-COV-2 positivity in 516 German intensive care and emergency physicians studied by seroprevalence of antibodies National Covid Survey Germany (NAT-COV-SURV)
Source: PLoS One. 2021 Apr 8;16(4):e0248813. doi: 10.1371/journal.pone.0248813 (PMC8031427; doi:10.1371/journal.pone.0248813)
Supplement: Attachment — Submitted filename: PONE-D-20-39396 Response to Reviewers.docx [file pone.0248813.s001.docx]

**PONE-D-20-39396 Response to Reviewers**

Dear Editor, Dear Reviewers,

we appreciate very much your comments which help us to improve this manuscript. We adressed all requirements as follows:

**To the Editor**

1) We checked the style requirements and renamed all files, if necessary
2) We provided a table (Table 3) which shows relevant results regardless of significance. We included p-values.
3) According to german federal and state jurisdiction (DGSVO, Datenschutzgrund-verordnung) data are protected and cannot be shared publicly. The approval by the committee of medical ethics at Heinrich-Heine University Duesseldorf (*2020-1034) did not allow transfer of data outside of the university. However, data can be made available upon request. For any data request researchers may contact: (1) Committee of Medical Ethics of Heinrich-Heine University, Medical Faculty Heinrich-Heine University Duesseldorf, Moorenstr. 5, D-40225 Duesseldorf; [ethikkommission@med.uni-duesseldorf.de](mailto:ethikkommission@med.uni-duesseldorf.de), or (2) the data protection commissioner of the university hospital: Datenschutzbeauftragter, University Hospital Duesseldorf, Moorenstr. 5, D-40225 Duesseldorf; [datenschutz@med.uni-duesseldorf.de](mailto:datenschutz@med.uni-duesseldorf.de)
4) We removed the funding information from the Acknowledgement section. There was no specific funding. All expenses including laboratory analysis were paid by regular institutional budget.
5) Dr. Ingo Mrosewski is employed by the MDI Limbach Berlin GmbH, a commercial clinical laboratory. The MDI Limbach GmbH did not play a role in the study design, data collection and analysis, decision to publish, or preparation of the manuscript. It did not provide specific financial support but all costs for laboratory analysis were paid for by the Dept. of Emergency Medicine at University of Herdecke. This affiliation does not alter our adherence to PLOS ONE policies on sharing of data and materials. All other authors have nothing to declare
6) We removed figure 1 from this submission. We considered presenting the data in a table. However, when thoroughly analysing the information, the figure as well as table would just show that participants came from almost all parts of Germany, with the highest number coming (as expected) from the most densily populated areas, and no relation between rate of positivity and region. We mentioned that in the results section stating that participants came from all over germany and naming the contribution oft he largest federal states. For the sake of brevity, we removed the figure withour adding a new table.
7) We included table 3 in the manuscript as well. There are no supplementary files.
8) The ethics statement has been removed from the acknowledgement section.

**Reviewer # 1:**

1) In the method section, it is not clearly mentioned which participants have been tested by RT PCR. While in the results section, it is stated that only 222 participants have been tested by RT PCR and/or antibodies. I think it should be more explicitly explained.

We clarified this aspect in the methods section (page 4):

*All participants answered a clinical questionnaire and from all participants 8 ml blood were drawn for SARS-CoV-2 antibody detection. All questionnaires and blood samples were given matching numbers and blinded. In some participants RT-PCR testing had been performed independent from this study. Participants were asked whether such tests had been performed and to provide the results of this tests, i.e. positive or a negative result.*

We clarified this aspect also in the results section (page 6):

*We found antibodies against the N protein of SARS-CoV-2 in 16/516 (3.1%) participants. SARS-CoV-2 tests via nasopharyngeal swab and RT-PCR and/or antibody detection independent from this study, i.e. at a time prior to participation in this study, had been performed in 222/516 (43%).*

2) Then there is a major published article on this issue that should be discussed in the discussion section. Since it included 10 034 healthcare workers in the UK and the comparison is interesting.

We added the article published by Eyre et al. and discussed it (Discussion, page 9)

*An interesting study with a large number of participants was published from the UK. Approx. 10.000 healthcare staff were tested voluntarily with RT-PCR and immunoassays for antibodies. The overall positivity was higher compared to our data, i.e. 11.2%. The authors provide a detailed analysis and in line with our results staff with a confirmed household contact had the highest risk for Covid-19 with an OR of 4.82. Interestingly, physicians faced a lower risk compared to other healthcare staff which is also in line with the relatively low rate of infection in our participants ^11^.*

**Reviewer #2:**

1. Results should be shown in table format for ease of reading and interpretation. Would be helpful to show (in table form) the seropositive rates in specific subgroups e.g. those who cared for COVID patients, those with a positive family member, those with hx of febrile respiratory tract infection.

   We added a table (table 3) showing these results, i.e. data for those with a household contact, a stay abroad, a feverish resp. infection. We added also data on positivity rate versus availability of personal protective equipment. However, 86 % of participants worked in high risk-areas and 73% had a confirmed contact with Covid-19 patients. Therefore, we believe that almost everybody had a contact to SARS-CoV-2 positive patients, either knowingly or not. In the light of the low positivity rate, we think it is not meaningful to show separate data for these subgroups.
2. The authors state that “…11/18 (61%) were aware of their infection”. However, only 2 participants had previous positive PCR tests…how did the remaining 9 participants know about their infection? Through symptoms alone?

Thank you for this point. Here, our manuscript was unclear. The two positive RT-PCR tests were mentioned by participants in whom antibody testing was negative. Thus, the previous RT-PCR tests detected these two particpants. In other participants, PCR tests were done as well but provided no additional information because these particpants were detected by our antibody testing. We clarified this aspect in the Abstract section and in the results section

Page 2

*We recruited 516 physicans from all parts of Germany, 445/516 (86%) worked in high risk areas, and 379/516 (73%) had treated patients with COVID-19. The overall positive rate was 18/516 (3.5%), 16/18 (89%) had antibodies against SARS-COV-2, another 2 reported previous positive RT-PCR results although antibody testing was negative.*

Page 6

*Of those with positive antibodies or a positive RT-PCR 11/18 (61%) were aware of their infection – mostly due to previous RT-PCR testing unrelated to this study - and 7/18 (39%) were not aware.*

3. Was there any association between treating COVID positive patients and Seropositivity?

see #1

4. “A febrile resp tract infection after Jan 1 occurred in 87/499 (17%)…….but only 4/87 of those with a febrile resp infection were SARS-CoV2 positive, while 14/18 of positive patients had no febrile resp infection”. Suggest re-word to:
87/499 (17%) participants reported a febrile resp tract infection after Jan 1st. Among these, <5% were seropositive for SARS Cov2.
Among the 16 participants who were SARS Cov2 positive, only 2/18 reported a previous febrile resp infection after Jan 1st.

DONE, page 7

5. Hx of vaccination against seasonal influenza is not really relevant here, and can be removed. (why would previous flu vaccination affect serology results?)

We agree that previous flu vaccination should not affect infection rate with SARS-CoV-2 or serology results in healthcare staff. However, it was easy to obtain this information and we think that the additional information is worth to be given to the readers for a number of reasons. We have discussed this part on page 11.

At present, there is an ongoing discussion on the relevance of vaccination. Surprisingly, in some countries including Germany a number of healthcare staff is hesitant to be vaccinated against SARS-CoV2 despite clear data which speak in favour of the vaccination. The same observation has been made in recent years regarding vaccination against seasonal flu and data here show large positive effects which can be achieved with higher vaccination rates. Therefore, in the light of this ongoing debate, we believe every positive aspect of vaccination is worth tob e mentioned. We would be delighted if this (small) part of the manuscript could be accepted.

6. A limitation of the study is the long period (Jan to Jun) that participants were required to recall their symptoms, contact histories etc. For eg, the low number (2/18) of positive participants who reported previous febrile illness may be due to the fact that many participants could not remember their illness if it occurred several months ago

We have mentioned this limitation in the Limitation section on page 12:

*Additional cases of SARS-CoV-2 infection might have been missed due to seronegativity, oligo- or asymptomatic disease presentation without RT-PCR testing or because the study period was rather long and some participants may not remember their illness if it occurred several months ago. .*

**Reviewer #3:**

Thank you very much for your assessment
